# Effects of Dentifrice Containing Hydroxyapatite on Dentinal Tubule Occlusion and Aqueous Hexavalent Chromium Cations Sorption: A Preliminary Study

**DOI:** 10.1371/journal.pone.0045283

**Published:** 2012-12-28

**Authors:** Peiyan Yuan, Xiaoqing Shen, Jing Liu, Yarong Hou, Manqun Zhu, Jiansheng Huang, Pingping Xu

**Affiliations:** 1 Laboratory for Oral Diseases, Guangdong Provincial Stomatological Hospital, Southern Medical University, Guangzhou, China; 2 Department of Stomatology, Zhujiang Hospital, Southern Medical University, Guangzhou, China; University of Toronto, Canada

## Abstract

In order to endow environmental protection features to dentifrice, hydroxyapatite (HA) was added to ordinary dentifrice. The effects on dentinal tubule occlusion and surface mineralization were compared after brushing dentine discs with dentifrice with or without HA. The two types of dentifrice were then added to 100 µg/ml of hexavalent chromium cation (Cr^6+^) solution in order to evaluate their capacities of adsorbing Cr^6+^ from water. Our results showed that the dentifrice containing HA was significantly better than the ordinary dentifrice in occluding the dentinal tubules with a plugging rate greater than 90%. Moreover, the effect of the HA dentifrice was persistent and energy-dispersive spectrometer (EDS) revealed that the atomic percentages of calcium and phosphorus on the surface of dentine discs increased significantly. Adding HA to ordinary dentifrice significantly enhanced the ability of dentifrice to adsorb Cr^6+^ from water with the removal rate up to 52.36%. In addition, the sorption was stable. Our study suggests that HA can be added to ordinary dentifrice to obtain dentifrice that has both relieving dentin hypersensitivity benefits and also helps to control environmental pollution.

## Introduction

Hydroxyapatite (HA) is the main component of human bones and teeth [1], [2]. Synthetic HA has become one of the hot topics for biological material research over the past few decades due to its good biocompatibility and bioactivity. Since HA has a crystal structure similar to human teeth, preliminary researches exploring the effects of HA in easing dentine hypersensitivity, remineralization of early enamel lesion, and whitening by adding HA to dentifrice have been reported in recent years [3]–[8].

Dentine hypersensitivity refers to the transient and severe pain arising from stimulation of exposed dentine with cold, heat, and mechanical pressure. Increase in human life expectancy at the same time increases the lifetime of teeth in the mouth. Many diseases, including physiological wear, enamel hypoplasia, wedge-shaped defects, and gingival recession, can lead to exposed dentine [9]. The prevalence of dentine hypersensitivity thus shows a clear upward trend all around the world. It was reported that the prevalence of dentine hypersensitivity was about 4%–57% in adults, and that the prevalence reached up to 60%–98% in patients with periodontal diseases [10].

Currently, the most widely accepted mechanism of dentine hypersensitivity is the hydrodynamic theory proposed by Brannstrom in 1963 [11]. According to the hydrodynamic theory, temperature, physical or osmotic pressure changes lead to fluid flow within the dentinal tubules, triggering nerve fibers in dental pulp, which causes pain. Therefore, occlusion of dentinal tubules with materials, reducing dentine permeability, and reducing or preventing dentin fluid flow due to external stimuli can achieve the goal of relieving dentine hypersensitivity [6]–[8].

As to environment protection, economic development is accompanied with increased emissions of heavy metals to the environment. Many investigations revealed that the concentration of heavy metal ions always remained a high level in municipal sewage [12], [13]. Discharge of municipal sewage containing heavy metals into the environment directly is one of the important reasons leading to pollution. It has become a major environmental issue faced by many countries that need to be addressed urgently [14].

There are a variety of methods to remove heavy metal ions from liquid, such as chemical precipitation, ion exchange, electrochemical method, sorption, reverse osmosis, and so on [15], [16]. Among them, sorption is widely used due to its advantages of low cost, high efficiency, and easy operation. In recent years, numerous studies have found that HA has a strong sorption capacity, and is an excellent adsorbent for the removal of heavy metal ions from polluted water or soil [17]–[19].

We thus raise the possibility of mixing HA with dentifrice so that in addition to its role in dental care, HA can also adsorb heavy metal ions from the environment after the liquid waste containing dentifrice enters the sewer after tooth brushing, reducing the environmental heavy metal pollution. If feasible, it undoubtedly makes our daily oral care behaviors important for environmental protection, which has important social and economic benefits and opens a new gate for the field of environmental protection.

## Results

### SEM observation of dentinal tubule occlusion by dentifrice

**Figure 1:**
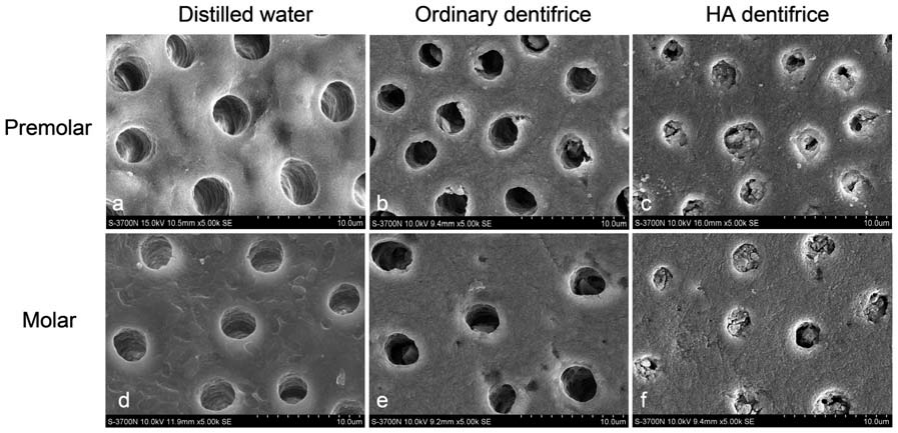
Dentinal tubule occlusion of dentine discs after treatment. (SEM, 5000×) (a, d) Dentinal tubules were empty without any occluding materials. (b, e) The majority of dentinal tubules were empty and a small number of dentinal tubules showed a sparse existence of occluding materials. The chunky occluding materials locate in the middle of the dentinal tubules with clear boundaries between the precipitate and dentin. (c, f) The majority of the dentinal tubules were blocked by materials. The occluding materials adhered to the dentinal wall with blurred boundaries between part of the precipitate and dentin.

The SEM observations of the premolar dentine discs after 7 days treatment of dentifrice were shown in Figure 1. The majority of the dentinal tubules were blocked by materials in the HA dentifrice group, while dentinal tubules were empty in the blank group and the ordinary dentifrice groups. Similar SEM observations were obtained for the molar dentine discs treated with dentifrice for 7 days as those for the premolar dentine discs.

### SEM observation of the persistence of dentinal tubule occlusion effects of dentifrice

**Figure 2:**
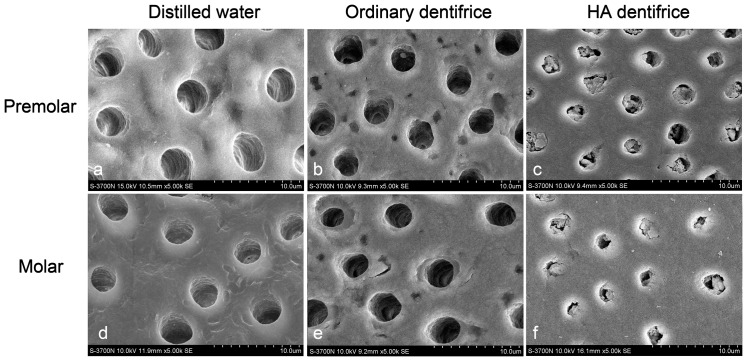
Dentinal tubule occlusion after mechanical brushing with distilled water. (SEM, 5000×) (a, d) Dentinal tubules were empty with no occlusion. (b, e) Almost all dentinal tubules were empty with very few dentinal tubules showing little occluding materials. (c, f) Most of the dentinal tubules showed various levels of sediment blockage.

Similar SEM observations were obtained for the premolars and molars dentine discs after treating the dentine discs with dentifrice for 7 days and then mechanical brushing with distilled water for extra 7 days (Figure 2). Most of the dentinal tubules still showed various levels of sediment blockage, although occluding materials were reduced after washing with distilled water.

### Dentinal tubule plugging rates

**Figure 3:**
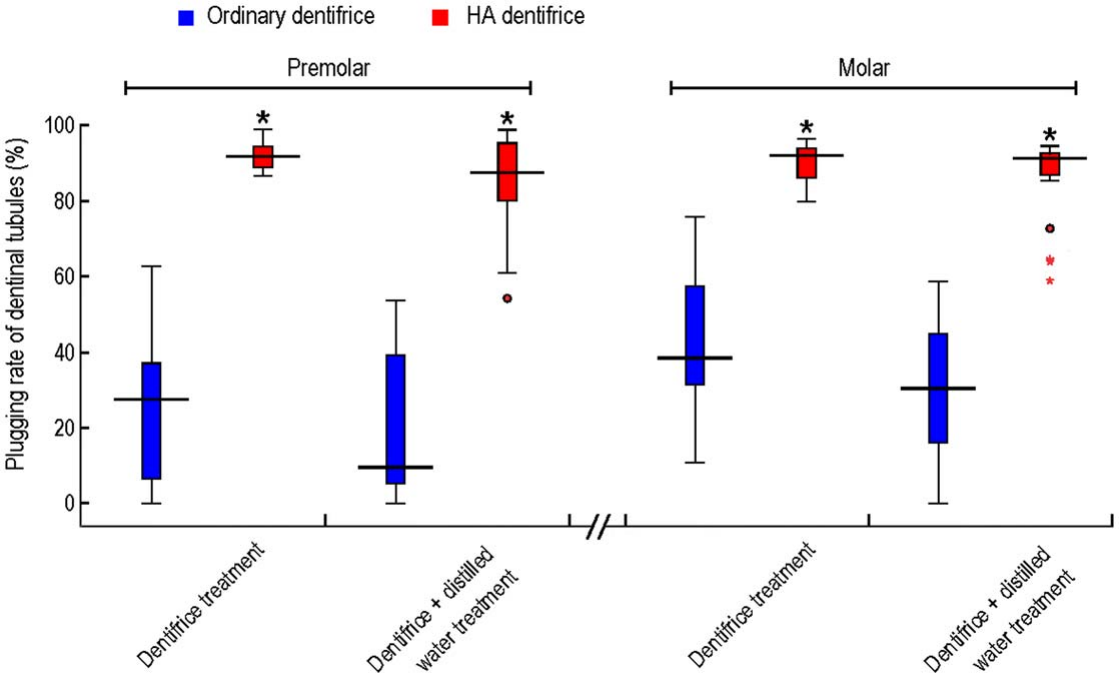
Plugging rates of dentinal tubules. The dentinal tubule plugging rate (*n* = 8) of the HA dentifrice group was significantly higher than that of the ordinary dentifrice group. *: *P*<0.01, compared with the ordinary dentifrice group.

The dentinal tubule plugging rates of the premolar dentine discs treated with dentifrice for 7 days were shown in Figure 3. The rate of the HA dentifrice group was significantly higher than that of the ordinary dentifrice group (*P*<0.001). Although the dentinal tubule plugging rates for the two groups were both slightly decreased after mechanical brushing with distilled water for 7 days, the rate of HA dentifrice group was still higher than the ordinary dentifrice group (*P*<0.001). Similar results were obtained for the molar dentine discs treated either with dentifrice for 7 days (*P*<0.001) or with distilled water for extra 7 days (*P*<0.001).

### Surface mineralization conditions of dentine discs

The major elements in each group detected by EDS include calcium (Ca), phosphorus (P), nitrogen (N), carbon (C) and oxygen (O). The groups with dentifrice treatment also had trace amounts of silicon (Si) and sodium (Na) that were used in friction modifiers. Ca and P elements, the main components reflecting the degree of mineralization, were analyzed statistically.

**Figure 4:**
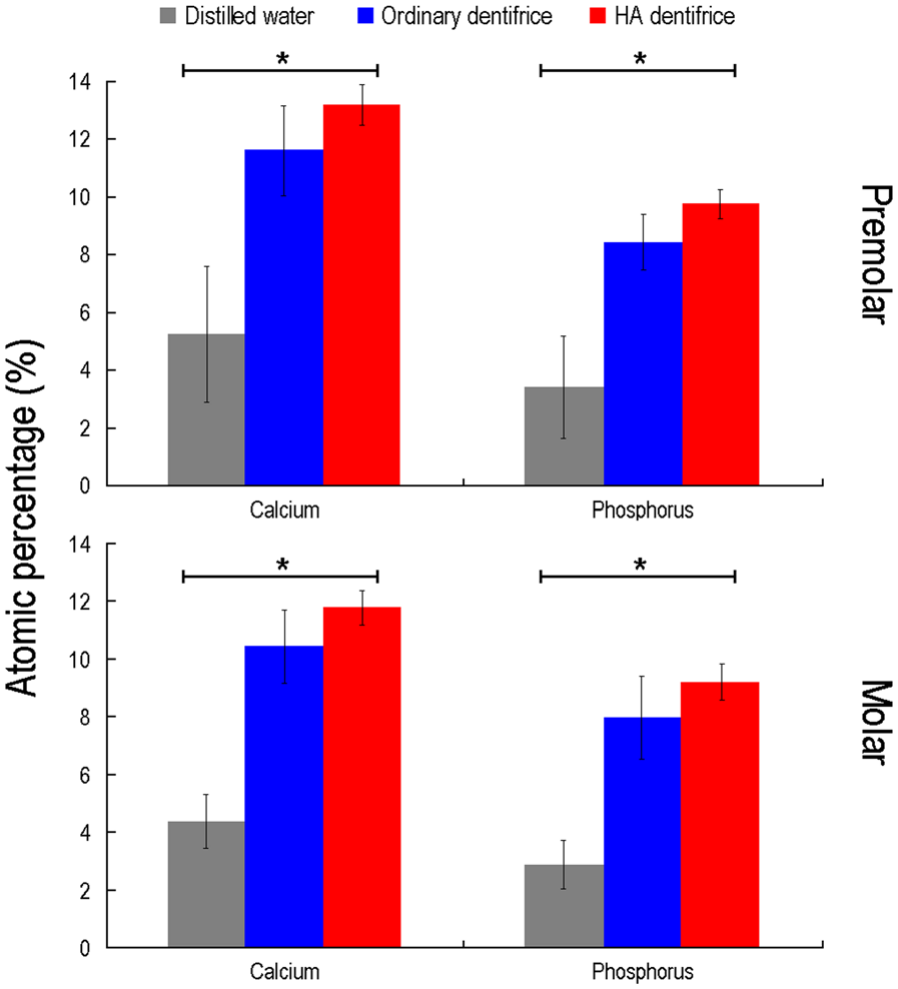
Atomic percentages of calcium and phosphorus in dentine discs. The lines on top of the bars stand for standard deviation. *: *P*<0.01, the differences of the atomic percentage (*n* = 8) of Ca or P among groups were statistically significant. Based on mean rank, the order of the atomic percentages of Ca or P is: HA dentifrice group>ordinary dentifrice group>distilled water group.

The test results for the atomic percentage of Ca in the premolar dentine discs were shown in Figure 4. The differences among groups were statistically significant (*P*<0.001). Similar results were obtained for the percentage of P for each group (*P*<0.001). Our results suggest that there were statistically significant differences in mineralization among various groups of dentine discs. Based on mean rank, it can be further inferred that the order of dentine mineralization degree is: HA dentifrice group>ordinary dentifrice group>distilled water group. The statistical results of Ca percentage (*P*<0.001) and P percentage (*P*<0.001) for the molar dentine discs were similar to those of the premolar dentine discs (Figure 4). Because the dentin discs of all three groups were stored in artificial saliva after brushing, the higher level of the atomic percentage of Ca and P in both dentifrice groups was resulted from brushing with dentifrice and the occluding dentifrice particles in dentinal tubules.

### Conditions of Cr^6+^ removal from water

**Figure 5:**
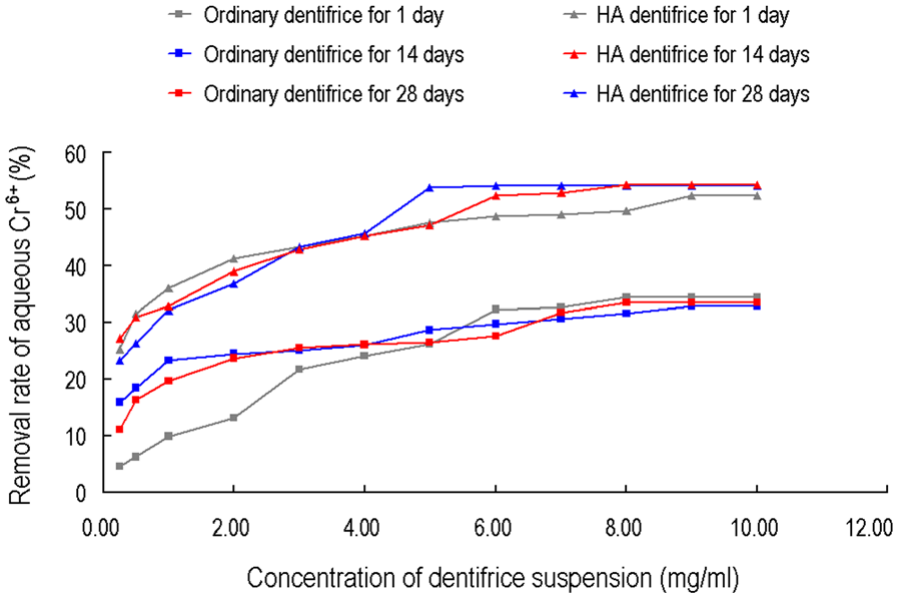
Concentration- and time-dependent removal rate of aqueous hexavalent chromium cations by dentifrices. The aqueous hexavalent chromium cations removal rate of the HA dentifrice group is always higher than that of the ordinary dentifrice group.

The Cr^6+^ concentration in water was gradually decreased with increasing amount of added dentifrice. The removal rate of the HA dentifrice group is always higher than that of the control. Cr^6+^ sorption of the HA dentifrice group did not show any obvious change with time. There were little changes when comparing the sorption curves after standing for 14 days or 28 days to that of standing for 1day. The ordinary dentifrice group showed unstable Cr^6+^ sorption. At low concentrations, the sorption capacities showed large fluctuations with the standing time, where no significant changes were observed for high concentrations (Figure 5).

**Figure 6:**
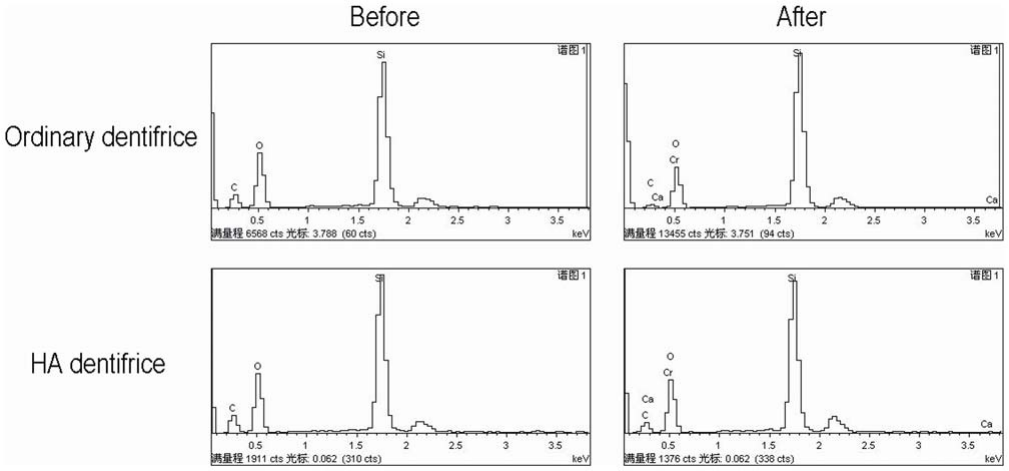
EDS spectra of dentifrice before and after sorption of chromium element. There is chromium element in the precipitates from both ordinary and HA dentifrice after sorption.

EDS detected the existence of chromium in the two groups of precipitates from dentifrice sorption (Figure 6), further confirming that Cr^6+^ in water was adsorbed and precipitated by dentifrice.

## Discussion

The in vitro dentine disc dentinal tubule blockage experiment has become the gold standard of dentine hypersensitivity [31]–[34]. Studies have proven that free liquids account for 75% of the dentinal tubule volume. The amount of fluid in the dentinal tubules is proportional to the fourth power of the diameter of dentinal tubules [35]. The surface characteristics of dentin determine 86% of the tubular fluid flow resistance [36]. Therefore, slight reduction of the dentinal tubule diameter can greatly reduce the flow of liquid within the dentinal tubules, subsequently alleviating the sensitivity of the nerve. Utilizing safe and effective biological materials through their physical or chemical properties to block exposed dentinal tubules, to reduce or inhibit the flow of tubular fluid, and to avoid stimulating pulp nerve endings is thus an effective means for controlling dentin sensitivities [37]–[39].

As seen from the SEM images, ordinary dentifrice with added HA showed a significantly increased effect of dentinal tubule occlusion after only 7 days of brushing. The effect is similar to commercially available anti-dentin sensitive dentifrices (data not shown) and that reported by the commercial researches of such products [40], [41]. The measured rates of dentinal tubule occlusion by the HA dentifrice were all above 90%, which were confirmed by dentine discs of the premolars and molars. Further studies focusing on the levels of surface mineralization of dentine discs demonstrated that Ca and P percentages on the surface of dentine discs treated with HA dentifrice increased significantly, suggesting that HA deposition and remineralization occurred on the surface of the dentine discs.

An outstanding anti-sensitivity dentifrice should exert continuous good occlusion effects of the dentinal tubules against all adverse external environments, thus achieving durable anti-sensitivity effects. It has been demonstrated that the mechanical brushing by toothbrush has strong abrasion effect on teeth [42], and that HA is soluble in solution, while saliva components can wash and dissolve on the sediments or mineralization products on dentin surface [43]–[45]. Our study thus designed a procedure of brushing with distilled water alone for 7 days after treated with dentifrice for 7 days. The results showed that although there was a slight loss of occluding materials after brushing with distilled water, the plugging rates of HA dentifrice for the dentinal tubules of premolars and molars were both above 85%, confirming that the occlusion of the dentinal tubules by the HA dentifrice is persistent.

In recent years, heavy metal pollution in waste water has become one of the major international environmental problems to be solved. Heavy metal ions in polluted water mainly consist of copper (Cu^2 +^), zinc (Zn^2 +^), lead (Pb^2+^), cadmium (Cd^2+^), nickel (Ni^2+^) and Cr^6+^, etc [27]–[30]. Previous studies mostly focused on the divalent metal ions, with few studies focusing on chromium ions.

Chromium is a cumulative poison with strong irritation and corrosiveness. It can invade the body through the respiratory tract, the gastrointestinal tract and the skin, which causes contact dermatitis and ulcers in the contact areas, chemical pneumonia, difficulty in breathing, liver damage, acute renal failure and other adverse reactions. Chromium poison presents longer duration and delayed healing [46], [47]. The toxicity of chromium is associated with its valence. Chromium in the environment mostly exists in two forms, Cr^6+^ and Cr^3+^ [48], [49]. The mobility and toxicity of Cr^6+^ are strong and Cr^6+^ has higher membrane penetration ability than Cr^3+^, thus more harmful to the human body. In addition, Cr^6+^ also has carcinogenic, reproductive toxic and mutagenic effects [50]–[53]. Therefore, Cr^6+^ is an important indicator for water pollution control.

This study found that adding HA to ordinary dentifrice significantly enhanced the sorption capacity of dentifrice for Cr^6+^ in water, with a removal rate up to 52.36%. In addition, all concentration groups showed stable sorption effects of Cr^6+^ without any significant fluctuations and desorption as the standing time increased, indicating that the HA dentifrice is a good Cr^6+^ adsorbent. Further analysis of the centrifuged precipitate after sorption via EDS test revealed that the powder contains chromium, confirming the role of dentifrice for the sorption of Cr^6+^. Since the sorption is stable, it can be inferred that the heavy metal ions adsorbed by HA and precipitated with the dentifrice particles is environmentally friendly.

As shown in the Cr^6+^ sorption curves of the HA dentifrice, the amount of adsorbed Cr^6+^ by the HA dentifrice initially increased with increasing amount of dentifrice. As the amount of dentifrice continued to increase, the sorption capacity plateaued and the sorption reached a balance. This hyperbolic saturation curve is consistent with the sorption isotherm curves obtained from previous studies using HA for the removal of heavy metal ions [17], [30], [54]. It suggests that the sorption process of Cr^6+^ by the HA dentifrice belongs to monolayer sorption, which discontinues once a monolayer is formed.

This is the first report on environmentally friendly dentifrice. We believe that environmentally friendly dentifrice refers to dentifrice with the dual functions of benefiting oral health and reducing environmental pollution. Our findings are not limited to the development of dentifrices. Based on the logic of this article, the philosophy of environmental protection can also be applied to the development of detergents used in our daily lives to form an industry of environmentally friendly commodities, thus adding more elements in harmony with nature to human life.

Our current study is only a preliminary step. In the future, we will focus on the HA concentration in the mixture, the particle size and surface modification of HA, and the sorption capacity of HA for multiple heavy metal ions mixture. We hope to continuously improve the effects of the HA dentifrice in occluding the dentinal tubules, while also achieving the goal of efficient sorption of a variety of heavy metal ions from water.

### Conclusions

Dentifrice containing HA could be effective in occluding the dentinal tubules and a good absorbent for Cr^6+^. It is a promising way to add HA in ordinary dentifrice to obtain a valuable product that has both relieving dentin hypersensitivity benefits and also helps to prevent heavy metal ions pollution.

## Materials and Methods

### Ethics Statement

The protocol was approved by the Academic and Medical Ethic Committee of Guangdong Provincial Stomatological Hospital, Southern Medical University (approval number: 2011014).

### Collection of isolated teeth

Twenty-four premolars and twenty-four molars with complete crowns and without decay, cracks, or previous reparative therapies that were extracted for orthodontic treatment or treatment of periodontal disease were collected after written informed consent. The teeth were cleaned of all surrounding soft tissues, scrubbed clean, and stored in neutral 10% formaldehyde solution for future use [20], [21].

### Preparation of dentine discs

The dentine disc preparation method improved by Mordan et al [22], [23] was used. Briefly, the occlusal enamel layer was removed by the use of turbine drill cooled with cold water. Dentine discs about 1.6 mm thick were obtained by cutting parallel to the occlusal surface 1.6 mm below the occlusal dentino-enamel junction. 600, 1000, 1200, and 2000 grit silicon carbide abrasive papers were used to stepwisely polish the occlusal side of dentine discs to a thickness of about 1.5 mm and to form a homogeneous, flat, and smooth surface. It was confirmed under a microscope that the samples were polished, with no scratches on the surface, and with homogenous transmittance. The polished samples were ultrasonicated in deionized water for 3 times in a row with 10 minutes each time in order to remove impurities, followed with rinsing with a large volume of deionized water for 3 times.

In order to remove the smear layer, dentine discs were etched with 10% citric acid for 120 seconds, washed with a large volume of deionized water, and ultrasonicated in deionized water for 3 times with 10 minutes each time. The dentinal tubules were exposed, thus generating dentine discs suitable for the research of dentine hypersensitivity.

### Preparation of dentifrice

The common calcium carbonate dentifrice and the HA/calcium carbonate dentifrice containing 3%(w/w) of HA with a particle size of 600 nm were made by the Foshan Engineering Center for Oral Care Products (Guangdong, China) according to national standards.

### Dentinal tubule occlusion

Twenty-four premolar dentine discs were randomly divided into 3 groups: the blank group treated with distilled water, the control group treated with calcium carbonate dentifrice, and the experimental group treated with HA/calcium carbonate dentifrice containing 3%(w/w) of HA. Each group contained 8 dentine discs.

Twenty-four molar dentine discs were similarly grouped and were used for parallel experiments.

Dentin discs were fixed on glass slides, and kept wet with distilled water. 0.2 g dentifrice was spread evenly on the entire dentin surface of the control group and the experimental group, and brushed with electric toothbrushes (Oral B, USA) at 7600 rpm and 100 g force for 2 minutes at room temperature. Dentin discs of the blank group did not receive dentifrice treatment, and distilled water was used instead. After standing for 2 minutes, dentine discs were rinsed with running water for 30 seconds to remove dentifrice on the surface. Slides were placed in 10 mL of artificial saliva containing Ca and P ions [24] and stored at a 37°C water bath. Fresh artificial saliva was replaced daily and the experiment was repeated 2 times daily for 7 days. Each dentine disc was then divided into 2 halves. One half was air dried, fixed with glutaraldehyde, sputtered with gold, and observed with scanning electron microscopy (SEM, S-3700N, Hitachi, Japan) under the condition of accelerating voltage of 10.0 kV and resolution of 3 nm. Three different regions from each sample were randomly selected for the determination of atomic percentages via energy-dispersive spectrometer (EDS). Area measurement was used with the following reading parameters: count rate is 2 CPS, reading time 100 s, and dead time 30%.

The other half of the dentine disc was brushed by electric toothbrushes with distilled water for 2 minutes each time and twice a day for extra 7 days, followed with SEM observation. The persistence of the dentinal tubule occlusion effects was then analyzed.

### Analysis of the dentinal tubule plugging rate

Based on the method of Ahmed et al [25], [26], the number of dentinal tubules (*N*) that were completely located in the SEM images were measured by the Image-Pro image analysis software (Media Cybernetics, USA). The scale of the software was calibrated to the scale bars in the images. The automatic measuring system of the software can automatically distinguish the boundaries of the dentinal tubules. The exposed areas of the tubules were selected and the total area of the selected region (*S*) was automatically calculated according to the scale. The average diameter of the exposed dentinal tubules in the blank group was used as the baseline (*D*). The plugging rate (*PR*) of the dentinal tubules in the dentine discs were calculated according to formula (1):
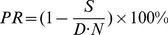
(1)


### Dentifrice sorption of aqueous hexavalent chromium cations (Cr^6 +^)

Potassium dichromate powder (K_2_Cr_2_O_7_) was dried in a 110°C oven for 2 hours. 2.83 g potassium dichromate was weighted and dissolved in 100 mL water. Five milliliters of the solution was diluted with distilled water to 500 mL and mixed well to prepare the 100 µg/mL Cr^6+^ solution.

The ordinary calcium carbonate dentifrice was taken as the control group and the HA/calcium carbonate dentifrice containing 3%(w/w) of HA was taken as the experimental group. Dentifrice suspensions of 0.25, 0.50, 1.00, 2.00, 3.00, 4.00, 5.00, 6.00, 7.00, 8.00, 9.00, or 10.00 mg/mL were prepared. One milliliter of dentifrice suspension of each concentration was added to 50 mL of the Cr^6+^ solution described above, stirred at 25°C and 300 rpm for 3 hours, and stand for 1 day, 14 days, and 28 days. After centrifuged at 3000 rpm for 10 minutes, 25 mL of the supernatants were taken, added with 3 mL of diphenylcarbohydrazide, and mixed well. After standing for 10 minutes, absorbance at 540 nm was measured by a UV spectrophotometer using distilled water as the reference solution. The precipitates after centrifugation were dried to powder in a 120°C oven, sputtered with gold, and the elements in the powder were detected by EDS [27]–[30].

The Cr^6+^ mass concentration in water was calculated according to formula (2):
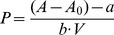
(2)where *P* is the Cr^6+^ concentration in water samples, *A* is the sample absorbance, *A*_0_ is the reference solution absorbance, *a* is regression equation intercept, *b* is regression equation slope, and *V* is mixture volume.

The Cr^6+^ removal rates by the dentifrice suspensions were calculated according to the formula (3):
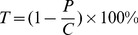
(3)where **T** is the removal rate, *P* is the Cr^6+^ concentration in water samples, and *C* is the initial concentration of Cr^6+^ solution.

### Statistic analysis

The SPSS16.0 (PASW, USA) package was used for statistical analyses. The difference in dentinal tubule plugging rates between dentine discs treated with ordinary dentifrice and HA dentifrice was examined using Wilcoxon-Mann-Whitney test. Wilcoxon-Kruskal-Wallis test was used to analyze the data for surface mineralization. The test level is α = 0.01.
